# Modulating Collagen I Expression in Fibroblasts by CRISPR-Cas9 Base Editing of the Collagen 1A1 Promoter

**DOI:** 10.3390/ijms26073041

**Published:** 2025-03-26

**Authors:** Karim Daliri, Jürgen Hescheler, Gregory A. Newby, Kendell Clement, David R. Liu, Kurt Pfannkuche

**Affiliations:** 1 Centre for Physiology and Pathophysiology, Institute for Neurophysiology, University of Cologne, Medical Faculty and University Hospital of Cologne, 50931 Cologne, Germany; 2 Marga and Walter Boll-Laboratory for Cardiac Tissue Engineering, University of Cologne, 50931 Cologne, Germany; 3 McKusick-Nathans Institute and Department of Genetic Medicine, Johns Hopkins University School of Medicine, Baltimore, MD 21218, USA; 4 Department of Biomedical Engineering, Johns Hopkins University, Baltimore, MD 21218, USA; 5 Institute for Nanobiotechnology, Johns Hopkins University, Baltimore, MD 21218, USA; 6 Department of Biomedical Informatics, University of Utah, Salt Lake City, UT 84108, USA; k.clement@utah.edu; 7 Merkin Institute of Transformative Technologies in Healthcare, Broad Institute of Harvard and MIT, Cambridge, MA 02142, USA; drliu@fas.harvard.edu; 8 Department of Chemistry and Chemical Biology, Harvard University, Cambridge, MA 02138, USA; 9 Howard Hughes Medical Institute, Harvard University, Cambridge, MA 02138, USA; 10 Center for Molecular Medicine Cologne (CMMC), University of Cologne, 50931 Cologne, Germany

**Keywords:** CRISPR, base editing, collagen, fibrosis

## Abstract

Fibrotic diseases, contributing to a significant portion of global mortality, highlight the need for innovative therapies. This study explores a novel approach to disrupt the expression of collagen by using adenine base editing to target Col1a1, a key gene driving both fibrosis and cancer metastasis. Editing Col1a1 in fibroblasts demonstrated 18% editing efficiency. An analysis of a specific clone harboring a CCAAT-to-CCGGA mutation in the Col1a1 promoter revealed reduced collagen production. Notably, when wild-type fibroblasts were cultured on the Col1a1-edited matrix, no compensatory collagen upregulation was detected, suggesting a lack of feedback mechanism in fibroblasts. Furthermore, the matrix derived from edited fibroblasts did not support the growth of MCF-7 cancer cells. These findings suggest that Col1a1 gene editing holds promise as a potential therapeutic strategy for fibrotic diseases. Further investigation is warranted to fully elucidate the implications of these findings for fibrosis and cancer.

## 1. Introduction

Fibrotic diseases, contributing to a substantial and escalating proportion of global mortality, represent a critical unmet clinical need [1]. These conditions, characterized by aberrant and progressive deposition of extracellular matrix (ECM), particularly collagen, lead to irreversible organ damage and, ultimately, organ failure [2]. The involvement of COL1A1, encoding a major component of collagen type I, is particularly evident in diseases such as systemic scleroderma, where its overexpression contributes to excessive collagen accumulation [3]. Furthermore, mutations in COL1A1 are central to the pathogenesis of Osteogenesis Imperfecta, characterized by fragile bones, and certain types of Ehlers-Danlos Syndrome, leading to connective tissue abnormalities and joint hypermobility [4,5]. Despite the prevalence and devastating consequences, current therapeutic interventions, often targeting downstream inflammatory pathways or ECM remodeling processes, have shown limited efficacy in modifying the underlying fibrotic processes [6]. Existing treatments, such as pirfenidone and nintedanib, have demonstrated only modest benefits and often come with significant side effects, highlighting the urgent need for more effective and targeted therapies [7]. Direct targeting of collagen synthesis, a central driver of fibrosis, remains an attractive, yet challenging, therapeutic avenue.

Collagen type I, the predominant fibrillar collagen in the ECM of most tissues, plays a crucial role in maintaining tissue architecture and mechanical properties. Excessive collagen deposition, mediated by COL1A1 and *COL1A2* (encoding the α1 and α2 chains of collagen type I, respectively), is a hallmark of fibrosis and directly contributes to impaired organ function. Dysregulation of COL1A1 expression, often driven by upstream transcriptional factors and epigenetic modifications, is a critical event in fibrotic pathogenesis [8,9]. Given the central role of COL1A1 in mediating collagen type I synthesis, we hypothesized that targeted suppression of COL1A1 expression could represent a promising antifibrotic strategy.

To achieve precise and durable modulation of Co1a1 expression, we leveraged adenine base editing (ABE), a cutting-edge gene editing technology that facilitates specific base conversions without introducing double-strand DNA breaks. ABE utilizes a catalytically impaired Cas9 variant fused to an adenosine deaminase, enabling the targeted conversion of adenosine to guanine (A→G) in genomic DNA [10]. This approach minimizes off-target effects compared to traditional CRISPR-Cas9 nucleases [11]. Specifically, we focused on the CCAAT box within the *Col1a1* promoter, a conserved regulatory element essential for transcriptional activation [12,13]. We reasoned that targeted base editing of the CCAAT box could disrupt transcription factor binding and reduce Col1a1 expression, thereby attenuating collagen synthesis and mitigating fibrotic processes.

## 2. Results

### 2.1. Design of a Gene Editing Strategy to Reduce Col1a1 Gene Expression

**Figure 1:**
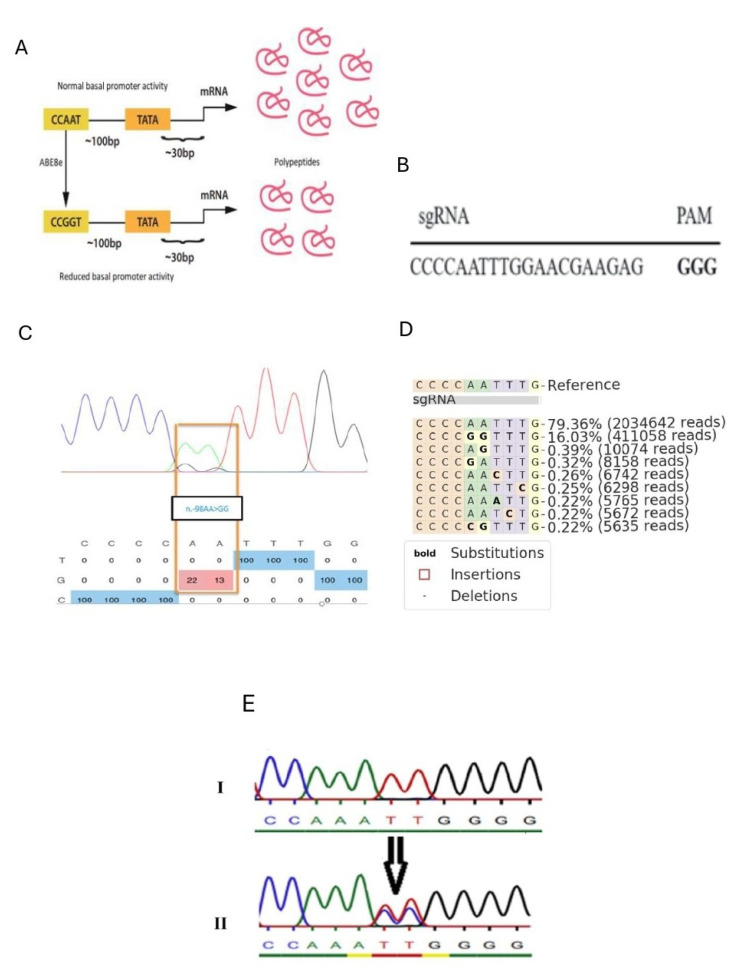
Editing of Col1a1 promoter inside fibroblasts. (**A**) Schematic of base editing strategy to target CAAT box. (**B**) A special sgRNA, which edited only AA in the CCAAT box thereby converting them GG upon. (**C**) Percentage of adenine (A) to guanine (G) editing in fibroblasts for each adenine in sgRNA following base editing with ABE8e and sgRNA, as determined by Sanger sequencing and EditR. (**D**) The outcome of adenine (A) to guanine (G) editing of DNA for each adenine along sgRNA in the fibroblasts treated with mRNA-ABE/plasmid-sgRNA, as determined by deep amplicon sequencing. (**E**) Sequencing results show the CCAAT box of the Col1a1 promoter before (I) and after editing (II). Each color represents a specific nucleotide base (A, T, C, G) in the DNA sequence.

ABE8e was employed to target the CCAAT box of the *Col1a1* promoter (Figure 1A). The 20nt protospacer was designed to use an optimal NGG PAM for S. pyogenes Cas9 and placed the “AA” of the target CCAAT box at positions 5 and 6 of the protospacer, the optimal substrate nucleotides for adenine base editing (Figure 1B). Substituting “AA” by “GG” may disrupt CBF transcription factor binding and hinder RNA polymerase II initiation for gene transcription.

Our gRNA effectively targeted the desired protospacer sequence within the Col1a1 promoter. Subsequent next-generation amplicon sequencing and Sanger sequencing demonstrated editing efficiency around 18% (Figure 1C,D). Following clonal expansion, multiple clonal cultures (*n* = 11) were genotyped by Sanger sequencing to identify those with successful editing and unedited controls. Using Sanger sequencing, we identified three clones with heterozygous disruptions in the CCAAT box, while the remaining clones harbored the wild type of genotype (Figure 1E).

**Figure 2:**
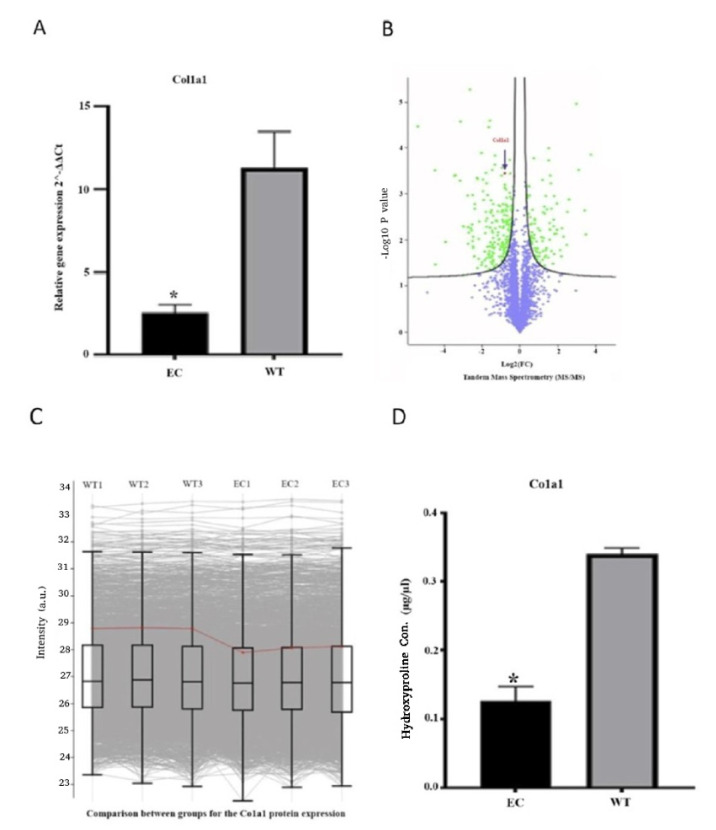
Comparison of *Col1a1 mRNA* and protein levels between wild-type (WT) and edited fibroblasts (EC). (**A**) *Col1a1 mRNA* relative expression was measured by qPCR, normalized to GAPDH, in wild-type (WT) and edited fibroblasts (EC) in triplicate. (**B**) Volcano plots illustrate differentially dysregulated proteins between EC and the WT control group. Among differentially expressed proteins, Col1a1 exhibited a significant downregulation. Log2-transformed fold changes are plotted against p values, derived from a two-sided unpaired Student’s *t*-test; *n* = 3 independent biological samples. (**C**) Profile plot displays Col1a1 protein abundance from the tandem mass dataset. The Col1a1 protein abundance trend from WT groups (WT1, WT2, WT3) to EC groups (EC1, EC2, EC3). The gradient indicates Pearson correlation to the centroid of each distribution, highlighted as a red line representing the trend of Col1a1 protein abundance. (**D**) Hydroxyproline content in edited cells (EC) and wild-type (WT) cells. The graph shows the hydroxyproline content in EC and WT groups. The WT group has a higher hydroxyproline content in comparison with the EC group (* *p*-value ≤ 0.05).

A quantitative PCR (qPCR) revealed a significant decrease in *Col1a1 mRNA* expression in ECs compared to WTs (Figure 2A). A profile plot generated from the tandem mass spectrometry (MS/MS) dataset revealed a reduction in Col1a1 protein abundance. This finding was observed across technical replicates, with comparisons between WT group and EC group demonstrating the consistent downregulation of Col1a1 protein (Figure 2B,C). We also confirmed a reduction in collagen secretion by measuring hydroxyproline in cell culture supernatants, which was consistent with *mRNA* and MS/MS results (Figure 2D).

### 2.2. Comparative Proteome Profiling on Fibroblast Adaptability to ECM Changes

**Figure 3:**
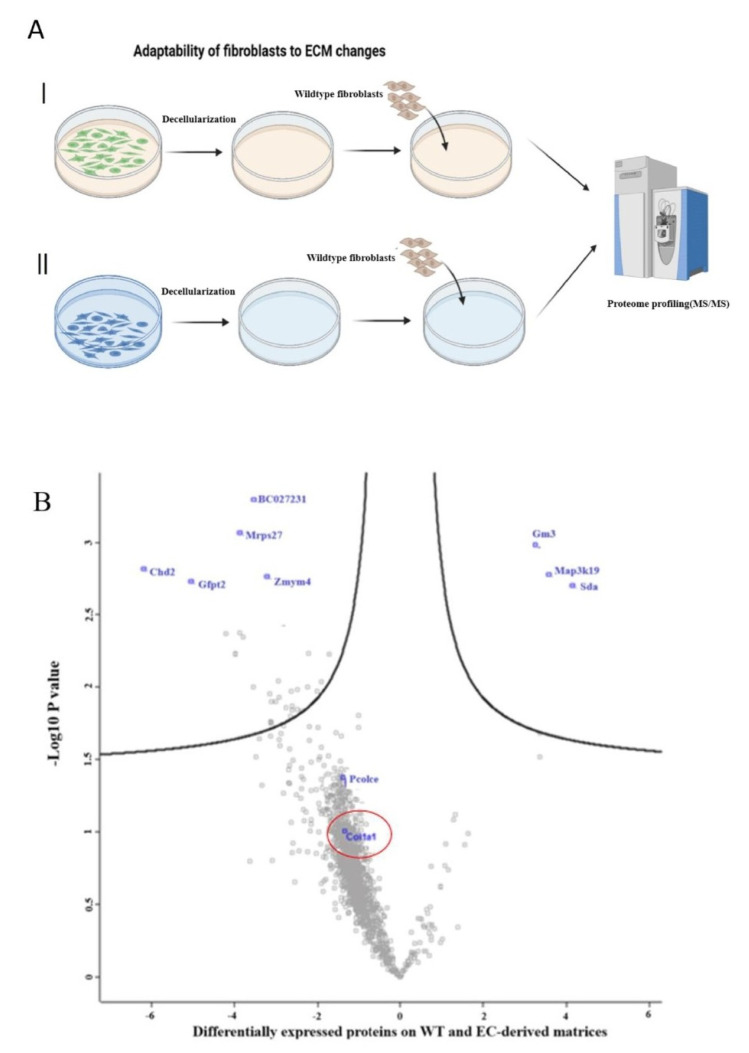
Fibroblast molecular and cellular behavior to the ECM derived from ECs. (**A**) Generating two ECM bioscaffold from WTs (I) and ECs (II) followed by analyzing WTs’ response using proteome profiling. (**B**) Wild-type fibroblasts exposed to decellularized ECM from ECs did not show significantly increased synthesis of Col1a1 and PCOLCLE proteins.

It can be hypothesized that WT cells would exhibit compensatory mechanisms in collagen synthesis when exposed to reduced collagen ECM. Indeed, given the challenges associated with precisely targeting all fibroblasts in vivo, WT cells may compensate for collagen turnover by increasing synthesis. To address this issue, we utilized cultured EC and WT cells in a standard two-dimensional (2D) cell culture to produce an ECM bioscaffold over a 5-day incubation period followed by decellularization. Subsequently, WTs were seeded onto the decellularized matrix for proteome profiling (Figure 3A).

A proteome analysis revealed that WTs do not exhibit increased synthesis of Col1a1 when exposed to the low-collagen ECM generated by ECs compared to the natural ECM secreted by WT cells. Procollagen C-endopeptidase enhancer 1 (*PCOLCLE* gene) also shows the same pattern of expression as Col1a1 (Figure 3B).

### 2.3. ECM from Collagen-Reduced Fibroblasts Suppresses Breast Cancer Cell Proliferation

**Figure 4:**
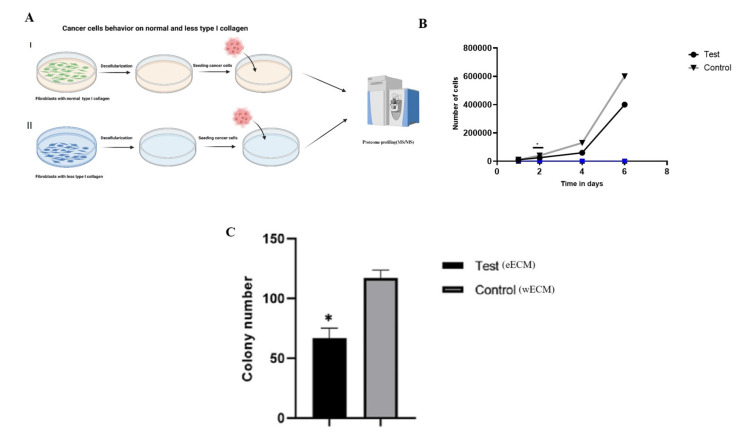
Reduced collagen density in the ECM alters MCF-7 cell behavior. (**A**) Experimental design for investigating the behavior of MCF7 cancer cells on normal and low-collagen matrices derived from WTs and ECs. (**B**) Cell proliferation assay results (the number of cells), as indicated MCF-7 cells were cultured in ECM derived from control (wild type) and test (edited cells) at the indicated time points. (**C**) Clonogenic assay (colony forming) assay results. The number of colonies between cells whose were cultured in ECM derived from control (wild type) and test (edited cells) (* *p*-value ≤ 0.05). The blue lines represent the standard deviation for each group, and the asterisk indicates a statistically significant difference between groups.

We examined the effect of ECM derived from endothelial cells (ECs) and wild-type fibroblasts (WTs) on mammary epithelial cancer cell (MCF-7) growth to explore potential links between the tumor microenvironment and cancer cell behavior (Figure 4A). We observed that MCF-7 cells cultured on ECM from ECs, which was characterized by lower collagen density relative to ECM from WTs, displayed reduced proliferation in cell proliferation assays (Figure 4B). Furthermore, clonogenic assays revealed an increased number of apoptotic cells in the MCF-7/eECM groups (Figure 4C), suggesting a possible link between the source and composition of the ECM and cancer cell survival.

## 3. Discussion

In this study, we efficiently targeted the CCAAT box upstream of the *Col1a1* gene in fibroblasts to introduce a precise small-nucleotide substitution (AA to GG). Engineering the CAAT box to reduce Col1a1 promoter activity has significant implications for gene regulation and cellular function. The modification leads to a marked reduction in both transcriptional and protein levels of COL1 in engineered cells. Interestingly, engineered cells exhibit an inability to compensate for the reduced activity of the Col1a1 promoter. This observation indicates that the cells cannot activate alternative pathways to compensate for COL1 reduction. Therefore, our approach provides a novel and innovative perspective for developing the next-generation antifibrotic treatment, based on targeting the Col1a1 promoter using a CRISPR-Cas9 adenine base editor.

The optimal strategy for mitigating or reversing fibrosis involves the removal or suppression of the underlying pathology. However, the elimination of the causative agent is not always practical. Moreover, a significant subset of patients shows suboptimal responses to conventional therapeutic agents resulting in advanced stages of fibrosis, which further complicates the treatment landscape with organ failure and death. In addition, there are only two approved drugs for fibrotic diseases, Nintedanib and Pirfenidone, which are both specifically used for patients with idiopathic pulmonary fibrosis (IPF). While these drugs exhibit the ability to slow the progression of the disease, neither has demonstrated effectiveness in reducing mortality, as indicated by recent findings. Moreover, a significant proportion of patients experience the need for treatment interruption due to severe side effects [14,15].

Our proposed strategy aims to mitigate or reverse detrimental conditions and thereby provide therapeutic benefits for fibrotic diseases. The concept of utilizing CRISPR-Cas9 to disturb the activation of deleterious pathways in fibroblasts can be adapted to various types of organs, including the liver, kidney, heart, lung, and skin, as fibroblasts are the primary producers of ECM proteins within fibrotic tissues and the tumor microenvironment [16]. By utilizing the CRISPR-Cas9 ABE8e base editing system, we achieved a permanent modification in the Col1a1 gene promoter, which is a key player in fibrosis development. While this permanent modification offers the potential for long-term suppression of Col1a1 in chronically fibrotic tissues, we believe that transient suppression of Col1A1 might be a more suitable therapeutic strategy in certain contexts [17]. In tissue under homeostasis, permanently altering Col1A1 expression could disrupt the delicate balance required for normal tissue function. Transient suppression strategies, such as using inducible CRISPR systems [18] or RNA interference [19], may offer a more controlled and reversible approach, facilitating a more nuanced modulation of collagen production. Our rationale for pursuing permanent modification stems from the chronic nature of fibrotic diseases [6], the potential for epigenetic compensation following transient suppression [20], and the possibility of precise fibroblast targeting to reduce potential side effects [21]. However, future research should directly compare the efficacy and safety of transient versus permanent Col1A1 suppression strategies to determine the optimal therapeutic approach for different fibrotic conditions.

The perturbation of Col1A1 may play a critical role in cancer. Following the disruption of tissue homeostasis due to oncogenic processes, a tissue repair response is initiated, which includes the activation of fibroblasts. This is followed by the development of a desmoplastic tumor stroma characterized by the excessive deposition of ECM components, notably collagens [22]. In some tumors such as breast cancer, the desmoplastic stroma comprises up to 90% of tumor mass and is associated with poor prognosis [23]. The ECM modulates the hallmarks of cancer; for example, increased collagen I density promotes mammary tumor initiation, growth, and invasion [24]. Thus, targeting ECM synthesis in stromal fibroblasts emerges as a potential strategy to limit cancer progression [25,26]. Our research suggests that exposing breast cancer cells to a new microenvironment with reduced collagen I leads to reducing cancer cell proliferation rate.

Furthermore, one important limitation to consider is the potential variability between fibroblasts derived from different organs or species. This heterogeneity could significantly impact research outcomes. To address this limitation, it is recommended to extend the approach to various fibroblast sources. NIH/3T3 fibroblasts were chosen for this study as they are considered a gold standard for primary collagen investigations, facilitating the optimization of the approach. However, future studies should incorporate fibroblasts from diverse organs and species to provide a more comprehensive understanding of fibroblast behavior and function. It is important to highlight that we propose the use of co-culture models (fibroblast–macrophage/epithelial cells) to investigate compensatory ECM collagen restoration across various signaling pathways, including TGF-β/CTGF signaling.

In conclusion, given the wide variety of affected organs, the chronic nature of the fibrotic processes, and the large number of individuals suffering devastating effects, these diseases pose some of the most serious health problems in current medicine. However, there remains a lack of widely accepted and effective treatments for these conditions [16]. So, our editing strategy could potentially contribute a universal solution to universal fibrotic problems as a molecular anti-fibrotic treatment with a permanent change at the DNA level with a potential to slow down or ideally reverse the fibrotic process. To further contextualize our findings and understand the broader implications of COL1A1 modulation, it would be beneficial to compare the observed effects of our ABE approach with the diverse impacts of COL1A1 elucidated through other established methods in various diseases, such as systemic sclerosis, osteogenesis imperfecta, and keloid formation. Future research is essential and should include evaluating the effectiveness of collagen promoter editing in comparison with currently available fibrosis medications, and the characterization of off-target editing from this approach. In vivo editing approaches in mice will also be essential to define the safety and efficacy of this approach in the context of disease models. Nevertheless, the findings described here point to an effective way to permanently reduce fibrotic load by genetic perturbation of an important transcription factor binding site that regulates COL1A1 expression.

## 4. Materials and Methods

NIH3T3 fibroblasts were obtained from Professor Gerhard Sengle Lab (University of Cologne, Cologne, Germany) and were cultured in Dulbecco’s Modified Eagle Medium (DMEM) (Thermo Fisher Scientific, Waltham, MA, USA), supplemented with 10% Fetal Bovine Serum (FBS) (Thermo Fisher Scientific, Waltham, MA, USA), and 1% penicillin/streptomycin (Thermo Fisher Scientific, Waltham, MA, USA). The fibroblasts were grown at 37 °C and 5% CO2 and routinely passaged using TrypLE™ Express (Thermo Fisher Scientific, Waltham, MA, USA) as a gentle trypsin replacement enzyme to detach and dissociate the cells.

### 4.1. Genotyping of the Col1a1 Promoter

Genomic DNA was extracted from fibroblasts using a DNeasy Blood & Tissue Kit (QIAGEN, Hilden, Germany). The DNA fragments spanning the CCAAT box region of *Col1a1* promoter were amplified using PCR with a pair of primers (forward, CACATCATGGCCCCTCCCTC; reverse, GAAGCCGAAGGCCAGCACG). The sequences of the PCR-amplified *Col1a1* promoter region were determined through Sanger sequencing (see Supplementary Figure S1 for details).

### 4.2. Guide RNA Design

The 20nt protospacer (CCCCAATTTGGAACGAAGAG) was designed to use an optimal NGG PAM for S. pyogenes Cas9 and placed the “AA” of the target CCAAT box at positions 5 and 6 of the protospacer, the optimal substrate nucleotides for adenine base editing. The designed gRNA was first synthesized. In the post-synthesis phase, the gRNA was efficiently ligated into the 132,777 plasmid (pU6-pegRNA-GG-acceptor, Addgene ID: 132777).

### 4.3. mRNA Production

ABE8e *mRNA* was a gift from Professor David Liu’s lab (the Broad Institute of Harvard and MIT, USA) and generated in D. Liu’s lab as follows.

ABE8e *mRNA* was transcribed in vitro from PCR products using full substitution of N1-methylpseudouridine for uridine. *mRNA* was capped co-transcriptionally using CleanCap AG analog (TriLink Biotechnologies, San Diego, CA, USA) resulting in a 5′ Cap 1 structure. The in vitro transcription reaction was performed using the NEB HiScribe T7 High Yield RNA Synthesis Kit (New England Biolabs, Ipswich, MA, USA) per the manufacturer’s instructions. A 120-base poly A tail was included in the primer and transcribed PCR product. *mRNA* was purified by lithium chloride precipitation, washed in 70% ethanol, and resuspended in nuclease-free sterile water. Purified mRNA was quantified using a NanoDrop spectrophotometer (Thermo Fisher Scientific, Waltham, MA, USA) and normalized to a concentration of 2 micrograms per microliter.

### 4.4. Transfection

Approximately 18–24 h before transfection, fibroblasts were seeded on 48-well plates at a density of 30,000–40,000 cells per well. To perform the transfection, TransIT-mRNA Transfection Reagent (Mirus Bio LLC, Madison, WI, USA) was used. The transfection mixture contained 1 μg ABE8e RNA, 500 ng of gRNA (plasmid), 2 μL TransIT-mRNA Transfection Reagent, and 2 μL Boost Reagent were added to 100 μL Opti-MEM medium (Thermo Fisher Scientific, Waltham, MA, USA), followed by a 5-min incubation at room temperature. Finally, the transfection complexes were added to the wells.

Approximately 72 h after transfection, genomic DNA (gDNA) was isolated with 75–150 μL of lysis buffer (10 mM Tris-HCl pH 8.0, 9.05% SDS, 25 μg·mL^−1^ proteinase K (Thermo Fisher Scientific, Waltham, MA, USA) at 37 °C for 1 h, followed by 80 °C for 30 min. In parallel with the transfection, clonal cell populations were isolated from the transfected cells using a limiting dilution approach. Single cells were spread into 96-well plates and allowed to form colony for around two weeks. Each individual clone was then screened using Sanger sequencing to identify and isolate isogenic cell lines carrying the desired mutation.

### 4.5. PCR Amplification and Sanger Sequencing

The relevant DNA fragment containing the mutations of interest was amplified using polymerase chain reaction (PCR). An amount of 50–100 ng of DNA was used as the starting material for all PCR amplifications. A PCR master mix was prepared using OneTaq Hot Start Master Mix with Standard Buffer (New England Biolabs, Ipswich, MA, USA), primers at 10 mM concentration (Invitrogen, Thermo Fisher Scientific, Waltham, MA, USA), and nuclease-free water (Synthego, Redwood City, CA, USA). The following primers were used for amplification: forward, GTCCCAGAAAGAAAGTACAAGGG; reverse, TGGAGAGCTGGGAGGAACC. The PCRs were purified using the PureLink™ PCR Purification Kit (Thermo Fisher Scientific, Waltham, MA, USA). The purified DNA was then subjected to Sanger sequencing (Eurofins Scientific, Ebersberg, Germany). For the sequencing reaction, 5 µL of primer was combined with 5 µL of PCR product.

### 4.6. High-Throughput Sequencing of Amplicons from Genomic DNA

The same DNA samples used in the Sanger sequencing method were subjected to high-throughput sequencing (Next-Generation Sequencing, NGS). The DNA fragments of interest were expanded and sequenced using an Illumina MiSeq device (Illumina Inc., San Diego, CA, USA) adhering to a previously established approach with minor modifications. Initially, a pair of primers (forward: ACACTCTTTCCCTACACGACGCTCTTCCGATCTNNNNCACATCATGGCCCCTCCCTC; reverse: TGGAGTTCAGACGTGTGCTCTTCCGATCTGAAGCCGAAGGCCAGCACG) with Illumina adapters was used to amplify the desired DNA segment during the first PCR cycle (PCR1). Next, a second PCR cycle (PCR2) was carried out using a primer pair containing a unique Illumina barcode and the unpurified PCR1 products as the template. PCRs were performed using a PrimeSTAR HS DNA Polymerase kit (Takara Bio Inc., Kusatsu, Shiga, Japan) following the manufacturer’s instructions. The PCR2 products were separated using 1.5% agarose gel electrophoresis, and the extracted bands were purified using a QIAquick Gel Extraction Kit (Qiagen, Hilden, Germany). Sequencing was then performed on an Illumina MiSeq instrument (Illumina Inc., San Diego, CA, USA) following the manufacturer’s protocol [27]. Sequencing reads were processed using MiSeq Reporter (Illumina Inc., San Diego, CA, USA), and amplicon sequences were aligned to a reference sequence using CRISPResso2 [28].

### 4.7. Quantitative-PCR (qPCR)

Quantitative-PCR (qPCR) was performed following a previously established protocol [29]. Fibroblast cells in culture were the source from which total RNA was extracted using the RNeasy Mini Kit (Qiagen, Hilden, Germany). From the extracted RNA, 1 μg was used as the template for synthesizing cDNA using the RevertAid First Strand cDNA Synthesis Kit (Thermo Fisher Scientific, Waltham, MA, USA).

Following synthesis, 1 μL from the cDNA was used for a 10 μL PCR amplification.The quantitative assessment of the threshold-cycle value was conducted using the Applied Biosystems™ PowerUp™ SYBR™ Green Master Mix (Thermo Fisher Scientific, Waltham, MA, USA) on an Applied Biosystems™ 7500 Fast Real-Time PCR System (Thermo Fisher Scientific, Waltham, MA, USA). In our experiment, GAPDH gene was used as the reference gene for normalization. Relative expression levels for the target genes were calculated using the 2-ΔΔCT method. The sequences of the qPCR primers used for amplifying *Gapdh* and *Col1a1* were as follows: *Gapdh-F,* CAGCCTCGTCCCGTAGACAA; *Gapdh-R,* CAATCTCCACTTTGCCACTGC; *Col1a1* -F, GTCCCAGAAAGAAAGTACAAGGG; and *Col1a1* -R, GATGGAGGGGCCGGACTCG.

### 4.8. Hydroxyproline Assay

A colorimetric assay kit (Sigma-Aldrich, St. Louis, MO, USA) was employed for measuring the content of hydroxyproline in the supernatant of cell culture plates by the manufacturer’s protocol. Initially, the supernatant of fibroblasts was subjected to hydrolysis using concentrated hydrochloric acid (HCl, ~12 M) at 120 °C for 3 h. To facilitate the evaporation of resulting fluid, plates were placed in a 60 °C oven for sample drying. Subsequently, 100 µL of the Chloramine T/Oxidation Buffer Mixture was added to each sample and standard well, followed by incubation at room temperature for 5 min. Next, 100 µL of the diluted DMAB Reagent (4-dimethylamino benzaldehyde) was added to each sample and standard well, with an incubation period of 90 min at 60 °C. To establish a standard curve, additional microplate wells were filled with known dilutions of hydroxyproline. The optical density of each well was measured at 560 nm. The hydroxyproline content in samples was subsequently determined by referencing the optical density values against the standard curve.

### 4.9. Protein Preparation for Liquid Chromatography-Tandem Mass Spectrometry (LC–MS/MS)

Samples were prepared as follows: an 8 M Urea/50 mM TEAB (Triethylammoniumbicarbonate) buffer was combined with a 50× protease inhibitor. Specifically, 20 µL of 50× protease inhibitors were added to 1 mL of 8 M Urea. One million cells were initially used. The cell pellet was lysed using the urea buffer. A Bioruptor (or an equivalent sonifier) was used with a cycle of 30/30 s. The sample was then centrifuged for 5 min at 20,000× *g*. Dithiothreitol (DTT) was added until the final concentration reached 5 mM, followed by a 60 min incubation at 25 °C. Chloroacetamide (CAA) was added to achieve a final concentration of 40 mM, followed by a 60 min incubation at 25 °C 60 min. Next, 50 mM TEAB was used to dilute the sample with a final urea concentration of ≤2 M. Trypsin was added with a substrate ratio of 1:75. Finally, samples were incubated at 25 °C overnight.

### 4.10. LC–MS/MS

All MS/MS experiments were performed at the CECAD Proteomics Facility (University of Cologne, Cologne, Germany) on a Q Exactive Plus Orbitrap mass spectrometer coupled to an EASY-nLC system (Thermo Scientific, Bremen, Germany). Peptides were loaded with solvent A (0.1% formic acid in water) onto an in-house packed analytical column (50 cm, 75 μm inner diameter, filled with 2.7 μm Poroshell EC120 C18 (Agilent Technologies, Santa Clara, CA, USA). Peptides were chromatographically separated at a constant flow rate of 250 nL/min using the following gradient: 3–5% solvent B (0.1% formic acid in 80% acetonitrile) within 1.0 min, 5–30% solvent B within 121.0 min, 30–40% solvent B within 19.0 min, 40–95% solvent B within 1.0 min, followed by washing and column equilibration. The mass spectrometer was operated in data-dependent acquisition mode. The MS1 survey scan was acquired from 300 to 1750 *m/z* at a resolution of 70,000. The top 10 most abundant peptides were isolated within a 1.8 Th window and subjected to HCD fragmentation at a normalized collision energy level of 27%. The AGC (Automatic Gain Control) target was set to 5 × 10^5^ charges, allowing a maximum injection time of 55 ms. Product ions were detected in the Orbitrap at a resolution of 17,500. Precursors were dynamically excluded for 25.0 s.

### 4.11. LC–MS/MS Data Analysis

All mass spectrometric raw data were processed with Maxquant (version 2.2 and 2.4) [30] using default parameters against the Uniprot canonical MOUSE database (UP589) and canonical HUMAN database (UP5640) with the match-between-runs option enabled between replicates. Follow-up analysis was performed in Perseus 1.6.15 [31]. Hits from the decoy database, the contaminant list and those only identified by modified peptides were removed. Afterwards, results were filtered for data completeness in replicates groups and LFQ values imputed using sigma downshift with standard settings. Finally, the false positive rate (FDR)-controlled *t*-tests between sample groups were performed with 0 = 0.2.

### 4.12. Preparation of ECM from Edited Fibroblasts

Matrices were prepared using a method similar to that reported previously [32]. Briefly, after culture for 1 week on DMEM/F-12, the cellular components were removed from the matrices through incubation with phosphate-buffered saline (PBS) containing 0.5% Triton X-100 and 20 mM NH_4_OH for 5 min at 37 °C. After the cellular components were removed by PBS washing, the surface was covered with PBS supplemented with 100 U/mL penicillin, 100 μg/mL streptomycin sealing with Parafilm^®^ strips, and stored for 2 to 6 weeks at 4 °C for further application.

### 4.13. Cell Proliferation and Clonogenic Assay

Cell proliferation and clonogenic assays were performed to assess the survival and proliferation potential of MCF7 cells on a new matrix with less collagen. Cell proliferation assays were performed as described previously [16]. Briefly, MCF7 cells were seeded at a density of 10,000 cells per six-well plate coated with ECM derived from isogenic fibroblasts. The cells were maintained in DMEM supplemented with 10% FBS. After the indicated time points, the cells in each well were trypsinized and counted to determine cell viability.

Clonogenic assays were used to assess the ability of MCF7 cells to form colonies. Cells were seeded onto 12-well plates at a density of 100 cells per well. Cultures were maintained for 12 days in complete culture media to facilitate the formation of colonies. The colonies were fixed with PFA 4% and stained with 0.5% crystal violet. Finally, the numbers of visible colonies containing at least 50 cells in size were counted.

### 4.14. Statistical Analysis

An unpaired Student’s *t*-test was used to analyze data between two groups. *p* < 0.05 was considered to indicate a statistically significant difference. Data is presented as the mean ± standard error of the mean. All analyses were performed using GraphPad Prism 10 (version 10.4.1, GraphPad Software, San Diego, CA, USA). For the network and gene ontology analysis, all statistical computations were performed using the corresponding packages.

## Data Availability

The data supporting the findings of this study are available from the corresponding author upon reasonable request.

## Supplementary Materials

The following supporting information can be downloaded at: https://www.mdpi.com/article/10.3390/ijms26073041/s1, Figure S1: Schematic of the Col1a1 promoter.

## References

[1] Rosenbloom J., Macarak E., Piera-Velazquez S., Jimenez S.A. (2017). Human fibrotic diseases: Current challenges in fibrosis research. Fibros. Methods Protoc..

[2] Herrera J., Henke C.A., Bitterman P.B. (2018). Extracellular matrix as a driver of progressive fibrosis. J. Clin. Investig..

[3] Abraham D., Lescoat A., Stratton R. (2024). Emerging diagnostic and therapeutic challenges for skin fibrosis in systemic sclerosis. Mol. Asp. Med..

[4] Infante A., Alcorta-Sevillano N., Macías I., Cabodevilla L., Medhat D., Lafaver B., Crawford T.K., Phillips C.L., Bueno A.M., Sagastizabal B. (2024). Galunisertib downregulates mutant type I collagen expression and promotes MSCs osteogenesis in pediatric osteogenesis imperfecta. Biomed. Pharmacother..

[5] Demirdas S., van den Bersselaar L.M., Lechner R., Bos J., Alsters S.I.M., Baars M.J.H., Baas A.F., Baysal Ö., van der Crabben S.N., Dulfer E. (2024). Vascular Ehlers-Danlos syndrome: A comprehensive natural history study in a Dutch national cohort of 142 patients. Circ. Genom. Precis. Med..

[6] Wynn T.A., Ramalingam T.R. (2012). Mechanisms of fibrosis: Therapeutic translation for fibrotic disease. Nat. Med..

[7] Galli J.A., Pandya A., Vega-Olivo M., Dass C., Zhao H., Criner G.J. (2017). Pirfenidone and nintedanib for pulmonary fibrosis in clinical practice: Tolerability and adverse drug reactions. Respirology.

[8] Devos H., Zoidakis J., Roubelakis M.G., Latosinska A., Vlahou A. (2023). Reviewing the Regulators of COL1A1. Int. J. Mol. Sci..

[9] Zheng Y., Khan Z., Zanfagnin V., Correa L.F., Delaney A.A., Daftary G.S. (2016). Epigenetic modulation of collagen 1A1: Therapeutic implications in fibrosis and endometriosis. Biol. Reprod..

[10] Tong H., Wang X., Liu Y., Liu N., Li Y., Luo J., Ma Q., Wu D., Li J., Xu C. (2023). Programmable A-to-Y base editing by fusing an adenine base editor with an N-methylpurine DNA glycosylase. Nat. Biotechnol..

[11] Neugebauer M.E., Hsu A., Arbab M., Krasnow N.A., McElroy A.N., Pandey S., Doman J.L., Huang T.P., Raguram A., Banskota S. (2023). Evolution of an adenine base editor into a small, efficient cytosine base editor with low off-target activity. Nat. Biotechnol..

[12] Saitta B., Gaidarova S., Cicchillitti L., Jimenez S.A. (2000). CCAAT binding transcription factor binds and regulates human COL1A1 promoter activity in human dermal fibroblasts: Demonstration of increased binding in systemic sclerosis fibroblasts. Arthritis Rheum. Off. J. Am. Coll. Rheumatol..

[13] Artlett C.M., Chen S.-J., Varga J., Jimenez S.A. (1998). Modulation of basal expression of the human α1 (I) procollagen gene (COL1A1) by tandem NF-1/Sp1 promoter elements in normal human dermal fibroblasts. Matrix Biol..

[14] Rogliani P., Calzetta L., Cavalli F., Matera M.G., Cazzola M. (2016). Pirfenidone, nintedanib and N-acetylcysteine for the treatment of idiopathic pulmonary fibrosis: A systematic review and meta-analysis. Pulm. Pharmacol. Ther..

[15] Henderson N.C., Rieder F., Wynn T.A. (2020). Fibrosis: From mechanisms to medicines. Nature.

[16] Rockey D.C., Bell P.D., Hill J.A. (2015). Fibrosis—A common pathway to organ injury and failure. N. Engl. J. Med..

[17] Manon-Jensen T., Kjeld N.G., Karsdal M.A. (2016). Collagen-mediated hemostasis. J. Thromb. Haemost..

[18] Zhang J., Chen L., Zhang J., Wang Y. (2019). Drug inducible CRISPR/Cas systems. Comput. Struct. Biotechnol. J..

[19] Wilson R.C., Doudna J.A. (2013). Molecular mechanisms of RNA interference. Annu. Rev. Biophys..

[20] Wang S., Zhang C., Hasson D., Desai A., SenBanerjee S., Magnani E., Ukomadu C., Lujambio A., Bernstein E., Sadler K.C. (2019). Epigenetic compensation promotes liver regeneration. Dev. Cell.

[21] Zhang X.-H., Tee L.Y., Wang X.-G., Huang Q.-S., Yang S.-H. (2015). Off-target effects in CRISPR/Cas9-mediated genome engineering. Mol. Ther. Nucleic Acids.

[22] Masugi Y. (2022). The desmoplastic stroma of pancreatic cancer: Multilayered levels of heterogeneity, clinical significance, and therapeutic opportunities. Cancers.

[23] Winslow S., Lindquist K.E., Edsjö A., Larsson C. (2016). The expression pattern of matrix-producing tumor stroma is of prognostic importance in breast cancer. BMC Cancer.

[24] Pickup M.W., Mouw J.K., Weaver V.M. (2014). The extracellular matrix modulates the hallmarks of cancer. EMBO Rep..

[25] Karamanos N.K., Piperigkou Z., Passi A., Götte M., Rousselle P., Vlodavsky I. (2021). Extracellular matrix-based cancer targeting. Trends Mol. Med..

[26] Venning F.A., Wullkopf L., Erler J.T. (2015). Targeting ECM disrupts cancer progression. Front. Oncol..

[27] Bronner I.F., Quail M.A. (2019). Best practices for Illumina library preparation. Curr. Protoc. Hum. Genet..

[28] Clement K., Rees H., Canver M.C., Gehrke J.M., Farouni R., Hsu J.Y., Cole M.A., Liu D.R., Joung J.K., Bauer D.E. (2019). CRISPResso2 provides accurate and rapid genome editing sequence analysis. Nat. Biotechnol..

[29] Schmittgen T.D. (2007). Quantitative gene expression by real-time PCR: A complete protocol. Real-time PCR.

[30] Tyanova S., Temu T., Cox J. (2016). The MaxQuant computational platform for mass spectrometry-based shotgun proteomics. Nat. Protoc..

[31] Tyanova S., Temu T., Sinitcyn P., Carlson A., Hein M.Y., Geiger T., Mann M., Cox J. (2016). The Perseus computational platform for comprehensive analysis of (prote) omics data. Nat. Methods.

[32] Franco-Barraza J., Beacham D.A., Amatangelo M.D., Cukierman E. (2016). Preparation of extracellular matrices produced by cultured and primary fibroblasts. Curr. Protoc. Cell Biol..

