# Supplementary material for: Modulating Collagen I Expression in Fibroblasts by CRISPR-Cas9 Base Editing of the Collagen 1A1 Promoter
Source: Int J Mol Sci. 2025 Mar 26;26(7):3041. doi: 10.3390/ijms26073041 (PMC11989027; doi:10.3390/ijms26073041)
Supplement: Supplementary file 1 [file ijms-26-03041-s001.zip › ijms-3522543-supplementary.pdf]

Supplement: primer binding site and position of edited nucleotides

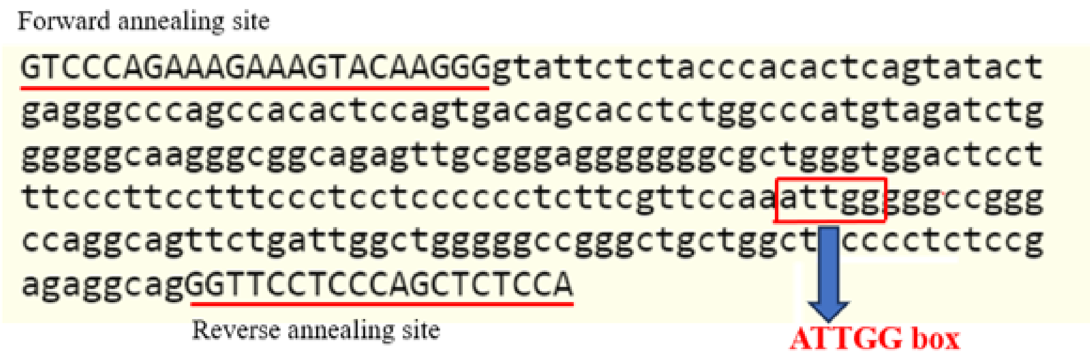

Supplementary Figure 1. Schematic of the *Col1a1* promoter. The image depicts the location of forward and reverse primers for amplification and targeted modification by prime editing, along with the regulatory ATTGG box. Targeting this region allows the modulation of collagen expression to reduce organ fibrosis.
